# Biochemical Response to Freezing in the Siberian Salamander *Salamandrella keyserlingii*

**DOI:** 10.3390/biology10111172

**Published:** 2021-11-12

**Authors:** Sergei V. Shekhovtsov, Nina A. Bulakhova, Yuri P. Tsentalovich, Ekaterina A. Zelentsova, Ekaterina N. Meshcheryakova, Tatiana V. Poluboyarova, Daniil I. Berman

**Affiliations:** 1 Institute of the Biological Problems of the North FEB RAS, 685000 Magadan, Russia; sigma44@mail.ru (N.A.B.); katusha@ibpn.ru (E.N.M.); dberman@mail.ru (D.I.B.); 2 Institute of Cytology and Genetics SB RAS, 630090 Novosibirsk, Russia; tanita11@mail.ru; 3 International Tomography Center SB RAS, 630090 Novosibirsk, Russia; yura@tomo.nsc.ru (Y.P.T.); zelentsova@tomo.nsc.ru (E.A.Z.); 4 Department of Physics, Novosibirsk State University, 630090 Novosibirsk, Russia

**Keywords:** freeze tolerance, freezing, cryoprotectants, glycolysis, Siberian salamander, *Salamandrella keyserlingii*

## Abstract

**Simple Summary:**

The Siberian salamander is a unique amphibian that is capable to survive long-term freezing at −55 °C. We used ^1^H-NMR analysis to study quantitative changes of multiple metabolites in liver and hindlimb muscle of the Siberian salamander in response to freezing. For the majority of molecules we observed significant changes in concentrations. Glycerol content in frozen organs was as high as 2% w/w, which confirms its role as a cryoprotectant. No other putative cryoprotectants were detected. Freezing resulted in increased concentrations of glycolysis products: lactate and alanine. Unexpectedly, we detected no increase in concentrations of succinate, which accumulates under ischemia in various tetrapods. Freezing proved to be a dramatic stress with high levels of nucleotide degradation products. There was also significant increase in the concentrations of choline and glycerophosphocholine, which may be interpreted as the degradation of biomembranes. Thus, we found that freezing results not only in macroscopical damage due to ice formation, but also to degradation of DNA and biomembranes.

**Abstract:**

The Siberian salamander *Salamandrella keyserlingii* Dybowski, 1870 is a unique amphibian that is capable to survive long-term freezing at −55 °C. Nothing is known on the biochemical basis of this remarkable freezing tolerance, except for the fact that it uses glycerol as a low molecular weight cryoprotectant. We used ^1^H-NMR analysis to study quantitative changes of multiple metabolites in liver and hindlimb muscle of *S. keyserlingii* in response to freezing. For the majority of molecules we observed significant changes in concentrations. Glycerol content in frozen organs was as high as 2% w/w, which confirms its role as a cryoprotectant. No other putative cryoprotectants were detected. Freezing resulted in ischemia manifested as increased concentrations of glycolysis products: lactate and alanine. Unexpectedly, we detected no increase in concentrations of succinate, which accumulates under ischemia in various tetrapods. Freezing proved to be a dramatic stress with reduced adenosine phosphate pool and high levels of nucleotide degradation products (hypoxanthine, β-alanine, and β-aminoisobutyrate). There was also significant increase in the concentrations of choline and glycerophosphocholine, which may be interpreted as the degradation of biomembranes. Thus, we found that freezing results not only in macroscopical damage due to ice formation, but also to degradation of DNA and biomembranes.

## 1. Introduction

Many northern amphibians are known to possess noteworthy adaptations to the environmental stress they encounter during the winter. The Siberian wood frog *Rana amurensis* is so far the only amphibian known to survive in the bottom of anoxic lakes for several months [1]. Several species overwintering on land are able to withstand deep freezing and the associated ischemic hypoxia [2]. These types of adaptations to extreme environmental conditions are expected to be based on similar metabolic foundations [3,4]. However, most studies were focused on a few pivotal metabolites; although comparisons of metabolomes would be promising, such studies on amphibians are still rare.

The Siberian salamander *Salamandrella keyserlingii* Dybowski, 1870 is unique among terrestrial tetrapods in its tolerance to long-term freezing. Berman et al. [5] demonstrated that adult animals may survive prolonged freezing at up to −50 °C (40% survival rate), and underyearlings, up to −55 °C (80% survival rate). Other freeze tolerant amphibians have significantly lower tolerance: Schrenk’s salamander *S. schrenckii* and the Japanese tree frog *Hyla japonica* from the south of the Russian Far East can survive up to −35 °C [6,7]. The wood frog *Rana sylvatica* of the Alaskan population, which was until recently believed to be the most freeze-tolerant frog, can withstand up to −16 °C or −18 °C [4,8], and the moor frog *R. arvalis* from West Siberia, up to −16 °C [9]. This remarkable freeze tolerance probably allowed *S. keyserlingii* to colonize vast territories of northern Eurasia, including the tundras along the shores of the East Siberian Sea at about 70° N.

Little is known on the mechanisms of freezing response in the Siberian salamander. The only study so far was performed in [10] on animals from the Kolyma populations, living in the continental part of the species’ range. Berman et al. [10] demonstrated that glycerol acts as a cryoprotectant in this species. Glycerol concentration in liver is <0.05% in fall; three-day-long freezing at −5 °C results in its increase to 2%; after gradual decrease to −10 °C in 20 days, to 7%; and by February, glycerol concentrations may be as high as 10–17% in liver and up to 18% in hindlimb muscles [10]. Freeze-tolerant animals are known to employ several ways of adaptation to ice formation [11,12,13]. These options include the synthesis of high concentrations of substances, such as sugars and polyalcohols, to lower the freezing point of water, or to employ ice-binding proteins that control ice growth.

The aim of this study was to investigate the biochemical response of the Siberian salamander to freezing and to compare it to that observed in other freeze-tolerant amphibians, as well as to the changes in the metabolome of the hypoxia-tolerant Siberian wood frog *R. amurensis*. Metabolomic data are currently available only for the latter species [14], enabling direct comparison.

We compared liver and hindlimb muscles of control (motile animals kept at 3–4 °C) and frozen ones, incubated for 7 days at −8 °C. Liver and muscles are routinely used in the studies on amphibian freeze tolerance [2,4,8], including the most relevant study on the *S. keyserlingii* [10]. Moreover, Siberian salamanders are very small animals and other organs would have to be pooled for a NMR study, so we had to limit ourselves to these organs. Quantitative data for 61 metabolites were obtained using proton nuclear magnetic resonance (^1^H-NMR). For most of these substances these are the first data obtained for frozen amphibians.

## 2. Materials and Methods

### 2.1. Animals Care and Freezing Protocol

Adult Siberian salamanders were caught in shoreline tundras on the northern shore of the Okhotsk Sea at about 59°40′ N in end August when the animals migrate from summers habitats (small water bodies) to overwintering sites. Animals were collected using approved methods under appropriate permits issued by cognizant governmental agencies (No. 001/05-20).

Fourteen individuals (mean weight ± SE, 4.9 ± 0.2 g; weight range, 2.6–8.8 g) were randomly allotted among the control and experimental groups. Seven individuals were exposed to freeze and other seven remained in the control group. Animals were kept in 250 mL plastic containers filled with moss with about 80% humidity, 2–3 individuals in each container. In the nature, Siberian salamanders do not feed since the migration to overwintering sites, so they were not fed during the experiment. The containers were gradually cooled according to the conditions observed in natural overwintering sites [15]: from 15 to 5 °C, in TSO-1/80 SPU thermostats (Tver, Russia); from 5 to 1 °C, in a WT-64/75 thermostat (Weiss Umwelttechnik GmbH, Leipzig, Germany) at 0.05° per hour [16] (Table 1). The control animals were further kept at 1 °C, while the experimental group was cooled to −8 °C (Table 1). Siberian salamander freeze at −2.5 to −4.5 °C [5], so the animals spent for two days at low negative temperatures (−2 and −3 °C) for acclimation. Given the potential variation in freezing temperatures, the salamanders could spend 10 to 13 days in frozen state, 7 of those at −8 °C. The temperature of −8 °C was chosen because it does not require prolonged cooling and provides guaranteed freezing at 100% survival rate [5].

After 7 days, the experimental animals reached −8 °C, and organs from both groups were quickly (20–30 s) extracted and immediately frozen in liquid nitrogen for extraction of metabolites.

### 2.2. NMR Measurements

Chloroform and methanol were purchased from Panreac (Spain, Barcelona); D_2_O 99.9%, from Armar Chemicals (Döttingen, Switzerland); all other chemicals, from Sigma-Aldrich (St. Louis, MI, USA). H_2_O was deionized using Ultra Clear UV plus water system (SG water, Hamburg, Germany) to the quality of 18.2 MOhm.

The extracts for NMR measurements were re-dissolved in 600 μL of D_2_O containing 2 × 10^−5^ M sodium 4,4-dimethyl-4-silapentane-1-sulfonic acid (DSS) as the internal standard and 20 mM deuterated phosphate buffer to maintain pH 7.2. The ^1^H-NMR measurements were carried out in the Center of Collective Use «Mass spectrometric investigations» SB RAS on a NMR spectrometer AVANCE III HD 700 MHz (Bruker BioSpin, Germany) equipped with a 16.44 Tesla Ascend cryomagnet as described in [17]. The proton NMR spectra for each sample were obtained with 64 accumulations. Temperature of the sample during the data acquisition was kept at 25 °C, the detection pulse was 90 degrees, and the repetition time between scans was 12 s. Low power radiation at the water resonance frequency was applied prior to acquisition to presaturate the water signal. The concentrations of metabolites in the samples were determined by the peak area integration respectively to the internal standard DSS.

The signal identification for the majority of metabolites was performed according to their NMR spectra available in literature [18] and in our in-house library [19] without additional confirmation. In cases when the signal assignment was unobvious, the identification was confirmed by spiking the extract with commercial standard compounds.

NMR spectra have been obtained for protein-free lipid-free extracts. For the smallest animals (four in the control and four in the experimental group) the amount of tissue (both liver and muscle) was insufficient, so organs from two individuals had to be pooled together to form one sample for analysis. We performed NMR analysis on four groups of tissues: muscle and liver samples from salamanders frozen at −8 °C, as well as from control animals. Each of those four groups consisted of five tissue samples representing independent replicates. Statistical analysis was performed on the MetaboAnalyst 5.0 web-platform (www.metaboanalyst.ca, accessed date: 11 November 2021) [20]. PCA scores and loading plots, Volcano plots, and heatmaps were constructed with the range data scaling to normalize the contributions of all metabolites [20]. Statistical significance of differences between the control and frozen groups was calculated using the Welch’s *t*-test.

## 3. Results

We analyzed the extracts of the liver and hindlimb muscles of the control and frozen Siberian salamanders. A total of 61 metabolites was identified (Table 2).

**Figure 1:**
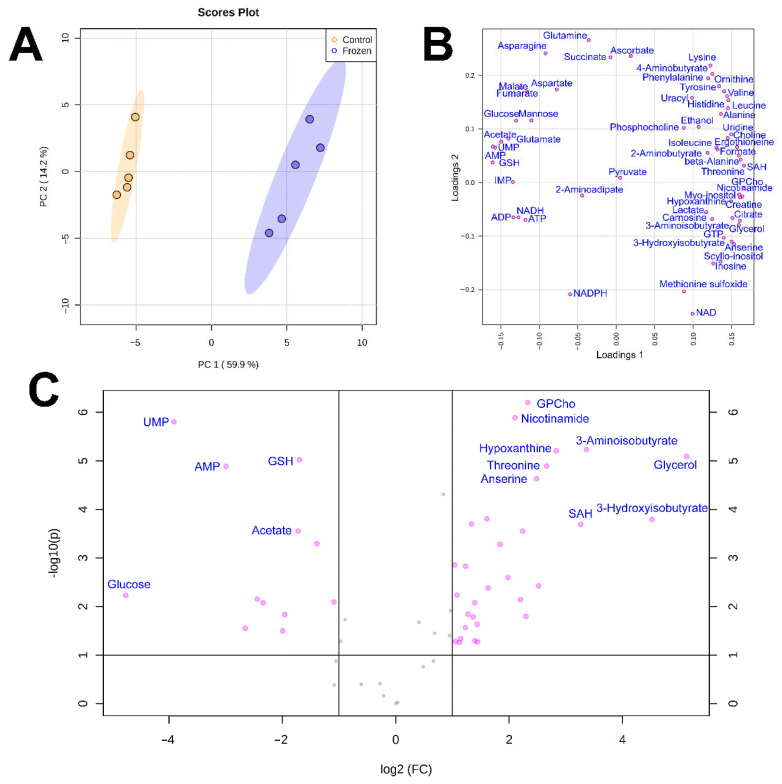
(**A**) Scores plot of the principal component analysis (PCA) of liver metabolomic profiles of frozen (blue) and control (yellow) individuals of *S. keyserlingii*. The data are range scaled. Colored ovals indicate 95% confidence regions. Variance explained by the first (PC1) and second (PC2) principal components is indicated on the axis of scores plot. (**B**) Loadings plot for the same data. (**C**) Volcano plot for frozen and control liver samples.

**Figure 2:**
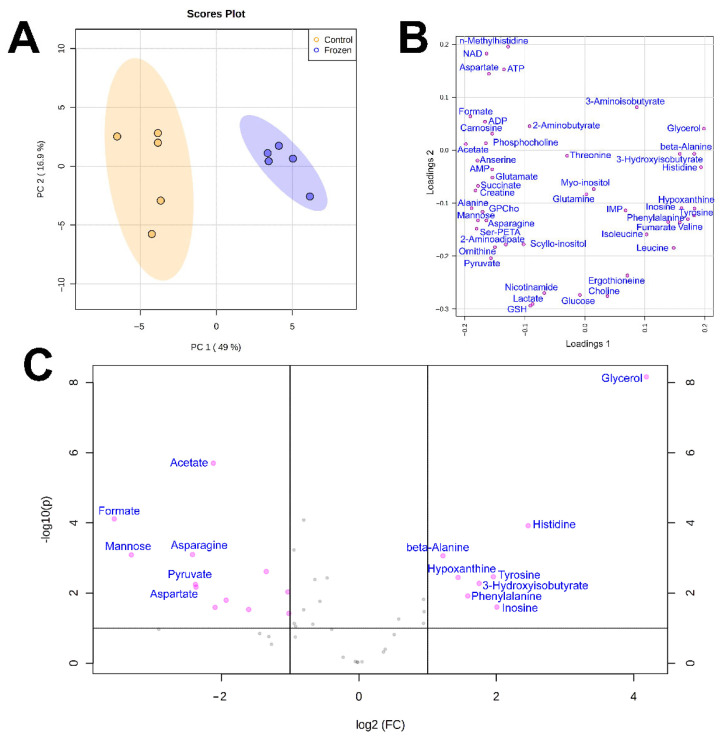
(**A**) Scores plot of the principal component analysis (PCA) of hindlimb muscle metabolomic profiles of frozen (blue) and control (yellow) individuals of *S. keyserlingii*. The data are range scaled. Colored ovals indicate 95% confidence regions. Variance explained by the first (PC1) and second (PC2) principal components is indicated on the axis of scores plot. (**B**) Loadings plot for the same data. (**C**) Volcano plot for frozen and control muscle samples.

The results of PCA analysis (Figure 1A or Figure 2A) demonstrated that the differences between the metabolomic compositions of tissues taken from frozen and control animals was significant. In loading plots, the majority of metabolites were arranged at the periphery of the graph, indicating that changes in concentrations were observed for most of them (Figure 1B and Figure 2B). This conclusion was supported by volcano plots (Figure 1C and Figure 2C).

The highest changes in liver were found for glucose, mannose, UMP, AMP, and IMP (decrease) and for glycerol, 3-OH-isobutyrate, hypoxanthine, S-adenosyl-homocysteine, nicotinamide, and glycerophosphocholine (increase). In muscle samples, the most significant decrease was observed for formate, mannose, acetate, asparagine, and pyruvate, and increase, for glycerol, histidine, tyrosine, 3-OH-isobutyrate, and inosine.

**Figure 3:**
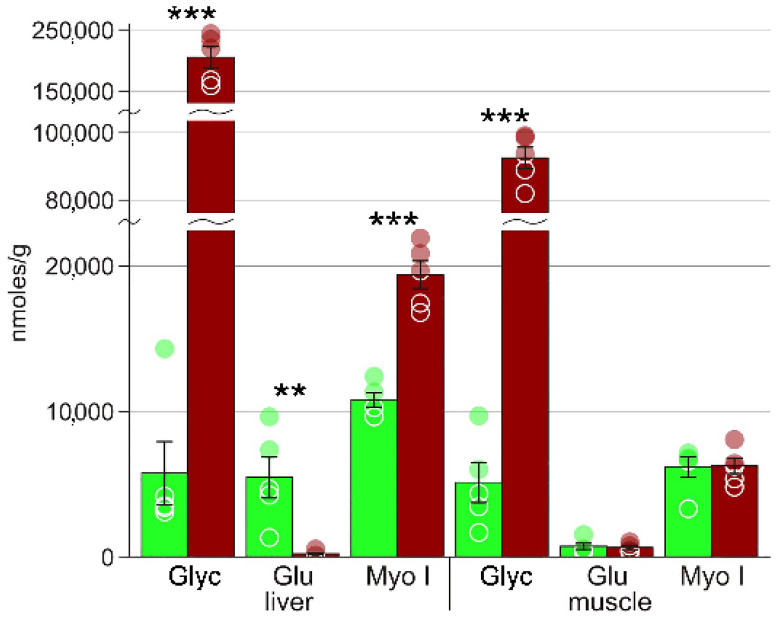
The concentrations of substances with cryoprotectant properties. Green columns, control; red, frozen; ** Welch test *p* < 0.01; *** *p* < 0.001; circles, individual data points; bar, SE. Glyc, glycerol; Glu, glucose; Myo I; myo-inositol.

**Figure 4:**
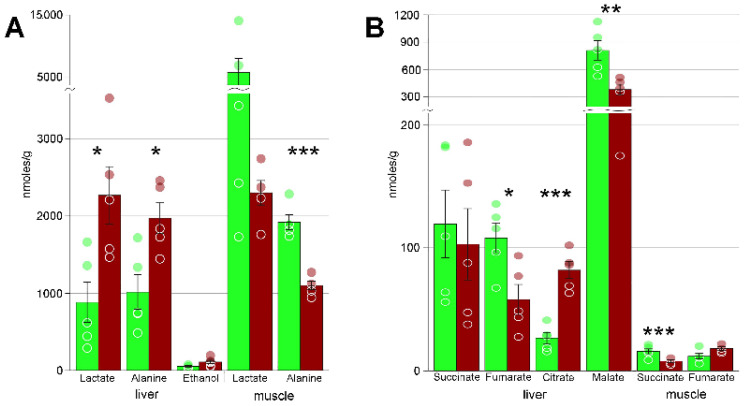
(**A**) concentrations of glycolysis end products; (**B**) Krebs cycle intermediates. Green columns, control; red, frozen; * Welch test *p* < 0.05; ** *p* < 0.01; *** *p* < 0.001; circles, individual data points; bar, SE.

**Figure 5:**
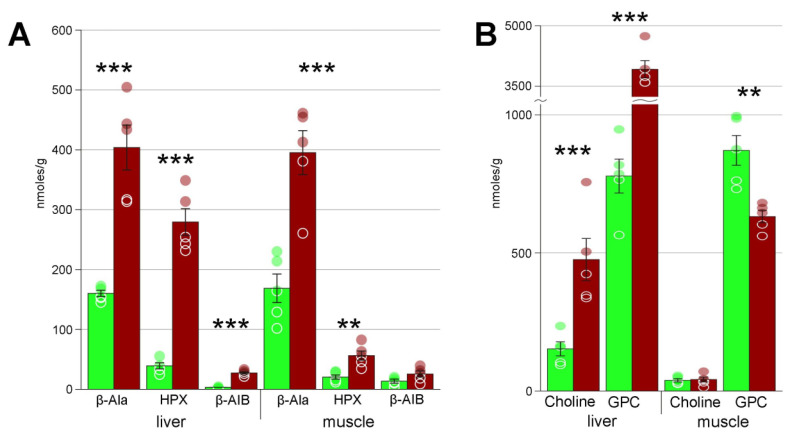
(**A**) products of nucleotide degradation: β-Ala, β-alanine; HPX, hypoxanthine; β-AIB, β-aminoisobutyrate. (**B**) Products of biomembrane degradation: GPC, glycerophosphocholine. Green columns, control; red, frozen; ** Welch test *p* < 0.01; *** *p* < 0.001; circles, individual data points; bar, SE.

**Figure 6:**
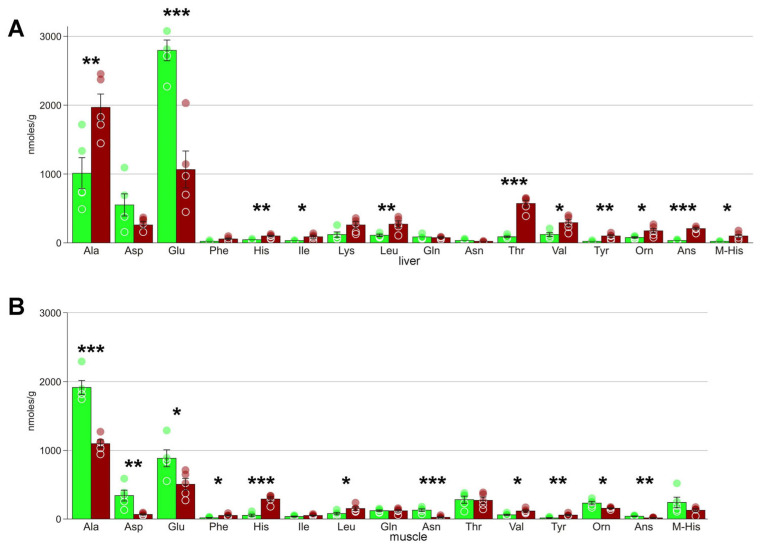
Concentrations of amino acids: (**A**) liver; (**B**) muscles. Green columns, control; red, frozen; * Welch test *p* < 0.05; ** *p* < 0.01; *** *p* < 0.001; circles, individual data points; bar, SE.

Heatmaps (Supplementary Figures S1 and S2) also demonstrated that concentration changes occur for virtually all metabolites; in liver, the decrease in concentration was observed for approximately one third of detected compounds, and the increase, for two thirds. In muscle, the numbers of metabolites with increased and decreased levels are approximately equal. In particular, we detected significant changes in several key metabolites that are probably central in the freezing response. These include the substances with cryoprotectant properties: glycerol, glycose, and myo-inositol (Figure 3). We also found several glycolysis end products: lactate, ethanol, and alanine, as well as certain Krebs cycle intermediates (Figure 4). Accumulation of large concentrations of nucleotide degradation products (Figure 5A) was observed in both liver and muscle in response to freezing. There was significant increase in membrane components, choline and glycerophosphocholine in the livers of the experimental animals, with a concomitant decrease in glycerophosphocholine in muscles (Figure 5B). We detected 13 proteinogenic free amino acids (Figure 6); their concentrations were mostly increased in frozen tissues.

## 4. Discussion

In this study we found that the content of the majority of the 61 identified metabolites significantly changed in response to freezing. For the majority of the substances these are the first data for amphibians, so we have nothing to compare them with. This makes our results not straightforward to interpret. Moreover, we estimate metabolite quantities as nmoles per gram of tissue, but one should be aware of the fact that freezing dramatically affects both the mass and composition of the whole body as well as of individual organs. According to Berman et al. [5], body mass decreases by about 1/4 during pre-freezing adaptation and freezing, mostly due to water loss. Liver size in frozen salamanders is drastically decreased, which is probably due to both water loss and glycogen depletion. Muscles also undergo significant water loss, which crystallizes as large ice crystals between the muscles and the skin. Therefore, the observed changes in metabolite concentrations may reflect both the changes associated with their synthesis, breakdown, or transport to other organs, and the result of water loss. However, we will make cautious interpretations of the most obvious of the obtained results.

### 4.1. Cryoprotectants

The remarkable tolerance of *S. keyserlingii* to freezing makes it a promising model object not just for amphibian physiology, but for the field of cryopreservation of cells, tissues and organs. There is a variety of mechanisms and molecules that are used for adaptation to freezing [21]. In this study we could observe only a set of small cryoprotective molecules.

Glycerol was the only observed low molecular weight cryoprotectant. Its average concentrations in frozen salamanders were 1.8% in liver and 0.8% in hindlimb muscles. Berman et al. [10] found that glycerol concentration in liver was 2% after a 3-day exposure to −5 °C, and 7% after 20 days of gradual temperature decrease to −10 °C. Our estimates were thus generally according to those from [10], but somewhat lower. This departure might be due to methodological differences, as wells as to the fact that the studied populations were from distinct regions: the highly continental climate of the inner Magadan oblast in [10] vs. cold monsoon climate the coast of the Okhotsk sea shore in this study. Significant differences in cryoprotectant accumulation are known to exist among populations of the freeze-tolerant frog *R. sylvatica* [8,22].

The concentration of free glucose in liver decreased approximately 30-fold. In the muscle, glucose concentrations remained unchanged, but were too low for the cryoprotective function (Figure 3). We should conclude that glucose is not a cryoprotectant in the Siberian salamander. In addition to glucose, we detected low levels of mannose in the liver, which were also highly reduced in frozen individuals (Table 2). We should note that we detected signals in the area around 5 ppm (doublets at 4.92 and 5.12 ppm; not shown) in frozen liver and muscles, which were undetectable in control samples. To our opinion they represent unidentified sugars that are formed in frozen tissues; their concentrations may be estimated as 5–10 and 1–3 nmoles/g, respectively.

Inositol, a cyclic sugar, is also a potential cryoprotectant [23]. In *S. keyserlingii*, we found high concentrations of myo-inositol in both organs (Figure 3), as well as another stereoisomer, scyllo-inositol, in the liver. The concentrations of myo-inositol were very high: about 6 μg/g in muscles and 10 μg/g in the liver of the control animals, which was more than of any other detected substance. Myo-inositol is known to be one of the main cryoprotectants in insects [24,25,26]. In the frozen liver of *S. keyserlingii* the average concentrations of myo-inositol increased twofold (Figure 3). However, that increased concentration of about 20 μg/g is still too low to be useful as a cryoprotectant (compared to hundreds of μg/g in the abovementioned insects). In vertebrates, myo-inositol is usually considered as a precursor of certain signal molecules [27], but the observed concentrations of this compound are obviously too high for that role. We believe that it is reasonable to hypothesize that the main function of myo-inositol in the Siberian salamander is osmolytic.

Multiple amphibian species can endure short-term freezing (for about a day at several degrees Celsius below zero), and several have more significant freeze tolerance [2]. These include *R. sylvatica* [28,29,30], *R. arvalis* [9,31], *Pseudacris crucifer* [32], *P. triseriata* [29,33], the closely related North American hylids, *Hyla chrysoscelis* [34,35] and *H. versicolor* [32], as well as the Asian *H. japonica* [7]. However, freeze tolerance of the abovementioned species is well below that of *S. keyserlingii* [2].

The cold tolerance of all studied ranids, as well as the hylids *P. crucifer* and *P. triseriata* is believed to be based mainly on glucose, with urea or glycolipids as less important cryoprotectants [2,8,28]. Organs and plasma of adult *H. chrysoscelis* and *H. versicolor* contain high concentrations of both glucose and glycerol [28,35,36,37,38,39]. Our study demonstrated that in the Siberian salamander, glucose or other sugars play no role in cryoprotection and it relies on glycerol alone. *S. keyserlingii* is thus so far unique among amphibians in its freeze tolerance strategy.

### 4.2. Freezing Bioenergetics

It is obvious that freezing highly reduces the rate of metabolic reactions. At the same time, it results in blood flow arrest and subsequent ischemia. Therefore, we might have expected to find certain substances associated with glycolysis and the markers of oxidative phosphorylation arrest in frozen organs.

Lactate and alanine are considered as the main glycolysis end products in amphibians [28]. Alanine is considered as the less toxic counterpart of lactate, because the accumulation of the latter results in acidosis [39]. The ratio of lactate to alanine is known to differ in different organs of the amphibians exposed to freezing [2,28]. Average concentration of lactate in the liver of the frozen Siberian salamander increased by 2.5 times (Figure 4A), and of alanine, by about two times. Moreover, in the liver we observed some amounts of ethanol, which is found as the major end product of glycolysis in some fish under anoxia [40]. Its content was on the average twice as high in frozen individual, but this increase was not statistically significant. Thus, the observed changes indicate upregulation of glycolysis in the liver of *S. keyserlingii*.

In the muscles of frozen salamander, however, the concentrations of lactate and alanine were on the average twice as low as in the control ones (Figure 4A). Moreover, their concentrations varied strongly among the animals within a group; these differences were statistically significant for alanine, but not for lactate. To explain this, we should note that the extremities are the first to freeze, and the concentrations of glycolysis end products in them should correlate with recent physical activity. The decrease in lactate and alanine would thus indicate decreased movement in freezing animals, and high variation would reflect how active the individual animals were prior to freezing.

As said above, freezing results in ischemia, which in our data is supported by increased concentrations of the end products of glycolysis. It would thus be reasonable to expect the suppression of oxidative phosphorylation. In vertebrates, suppression of oxidative phosphorylation is believed to result in the accumulation of high quantities of succinate, which is caused by the reversal of the activity of succinate dehydrogenase in the absence of oxygen [41]. Accumulation of succinate under hypoxia was detected in the Siberian wood frog *Rana amurensis* [14], the crucian carp *Carassius carassius* [42], and the red-eared slider turtle *Trachemys scripta* [43]. Surprisingly, we found no changes in succinate level in the liver, while in the muscle its average concentrations even decrease approximately twofold (Figure 4B). It could be due to the fact that ischemia is not so profound, or the Siberian salamander stops the Krebs cycle at a different point. Patterns in the liver favor the latter: the cycle appears to be arrested with the accumulation of citrate. The observed concentrations of malate are too high compared to other members of the cycle, so we believe that the majority of this substance in the liver was formed by some other biochemical process.

### 4.3. Markers of Stress

Freezing is obviously a harmful state even for the most resilient organisms. The forming ice can physically damage cells. Berman et al. [10] reported that some blood vessels of Siberian salamanders exposed to −35 to −40 °C were ruptured, which led to bleeding after thawing. However, we found that in addition to large-scale damage there is significant cellular stress. This is suggested by changes in concentrations of several metabolites. We detected a dramatic increase in the concentrations of hypoxanthine, β-alanine, and β-aminoisobutyrate, which are the degradation products of adenine, cytosine/uracil, and thymine (Figure 5A), as well as of inosine and inosinate in muscle (Table 2). Correspondingly, adenylate pools (ATP, ADP, and AMP) were reduced in both tissues (Table 2). This suggests that freezing induces high degree of nucleotide damage.

Glycerophosphocholine (GPC) and choline were found to significantly increase in liver in response to freezing; on the contrary, the concentration of GPC decreased in the muscle (Figure 5B). Choline is the precursor of acetylcholine and GPC acts as an osmolyte [44]; however, we believe that in this context these changes reflect their role as the components of phosphatidylcholines. We hypothesize that the observed changes in the concentrations of this compound reflect the breakdown of biomembranes and their transport to the liver for further degradation. Increased levels of choline and GPC in the liver thus also reflect the increased metabolic stress.

It thus turns out that freezing is a highly stressful condition. This is in apparent contrast to hypoxia in the Siberian wood frog *R. amurensis*. A study using the same methods [14] revealed reduced ATP/ADP ratio but no products of nucleotide degradation in response to aqueous hypoxia in this species, and the levels of GPC and choline were mostly reduced, not elevated. It looks like that the observed changes are well tolerated, since −8 °C is far from critical for *S. keyserlingii*. We should note that the observed changes in metabolite concentrations are measured in a tissue sample that may contain both damaged and intact cells, so it may be possible that the former heavily contribute to the pool of the observed nucleotide degradation products, while in intact cells the conditions are milder.

Significant reduction in GSH concentrations in both organs also might be a sign of stress (Table 2). In liver, we observed increased concentrations of ergothioneine and anserine, which are believed to act as weak antioxidants [45,46]. In muscles we detected ergothioneine, anserine, and carnosine, but their concentration either decreased (for anserine) or remained at the same level.

### 4.4. Amino Acids

Average concentrations of the majority of detected proteinogenic amino acids increased (although this increase was not always statistically significant). Similar observations were made for tolerant amphibians exposed to freezing [47] and hypoxia [14]. We believe that the most probable explanation of this increase is the arrest of protein synthesis during freezing. On the other hand, Niu et al. [47] note that many of the amino acids might act as cryoprotectants. However, amino acids detected in this study were found in much smaller concentrations (two to four order of magnitude lower) compared to glycerol, and thus their cryoprotective role is unlikely.

However, the concentrations of three amino acids, asparagine, aspartate, and glutamate, decreased (Figure 6). These changes could indicate profound shifts in particular cellular processes. First, these three amino acids contribute amino groups that go into the urea cycle, which might indicate at its activation. This is supported by the fact that in liver we detected higher levels of ornithine, which is also intermediate in the urea cycle. The methods used do not allow us to determine urea concentrations, so this hypothesis remains tentative. Another option is the reverse transamination reaction, in which aspartate and glutamate contribute their amino group with the formation of other amino acids and oxaloacetate or α-ketoglutarate, respectively. This might be supported by the increase of concentrations of other amino acids, but the reason of this reverse is unclear.

Noteworthy, Costanzo et al. [16,48] detected a significant increase in free aspartate and glutamate in *R. sylvatica* exposed to freezing preconditioning, as well as increased activity of glutamate dehydrogenase. They suggested that this is aimed at increasing the concentration ammonium ions resulting from glutamate deamination in order to accelerate ureagenesis. In the Siberian salamander, we see the opposite patterns of these molecules, so it may indicate that *S. keyserlingii* employs a totally different strategy for freezing response than *R. sylvatica*.

Anserine and 3-methylhistidine were the two amino acid derivatives with significantly changed concentrations. Anserine is a buffering agent and an antioxidant [49]. Its concentrations significantly increased in liver while decreasing in muscle (Figure 6). The closely related carnosine that has similar functions was found in muscle, where it also demonstrated a decreasing trend, which was not statistically supported (Table 2). 3-methylhistidine is a marker of muscle breakdown [50]. Its concentrations were significantly increased in liver, but the changes in muscle were not statistically significant.

### 4.5. Comparison to Metabolome in Hypoxia

So far, there are very few papers on the metabolomes of frozen amphibians. Niu et al. [47] studied the metabolome of the frozen *Nanorana parkeri*, but this species is not freeze-tolerant, and the obtained metabolites almost do not overlap with our data. Fortunately, we have got the results obtained for the Siberian wood frog *R. amurensis* using the same protocol [14]. For that species, we have the data for the heart and the liver, so we may directly compare the results only for the latter organ.

One unexpected finding was that freezing was much more stressful. In the Siberian wood frog, the amount of ATP decreased, and that of ADP and AMP increased, but the combined adenosine pool remained the same. On the contrast, in the Siberian salamander concentrations of all adenosine phosphates decreased dramatically with the appearance of products of their degradation.

As stated above, freezing effectively causes ischemia, and we found certain glycolysis upregulation n the Siberian salamander that is a feature of hypoxia, but no succinate accumulation. The latter might be explained by the fact that low levels of oxygen consumption continue even in frozen state, as shown by Voituron et al. [31] for *R. arvalis*.

Differences were also found in the concentrations of amino acids. In the hypoxic Siberian wood frog we observed elevated concentrations of all free amino acids except for aspartate, while in the liver of frozen Siberian salamander the concentrations of three amino acids were decreased: glutamate, aspartate, and asparagine (for the latter two, the average concentrations decreased two-fold, but this was nonsignificant due to high variance) (Figure 6). This is of high importance, because glutamate is the most abundant amino acid in the liver of non-frozen salamander; it is used not just for making proteins but participates in many crucial biochemical processes (Section 4.4). Therefore, it turns out that despite the alleged similarity of freezing and hypoxia, certain central processes run in opposite directions.

Another discrepancy is that the concentrations of choline and glycerophosphocholine significantly increase in the liver of Siberian salamander but decrease in the Siberian wood frog. This similarly implies different directions in the remodeling of biological membranes.

Therefore, the comparison of metabolomes of frozen *S. keyserlingii* and hypoxic *R. amurensis* indicates that these two states share the upregulation of glycolysis but otherwise differ in many important aspects that are yet to be elucidated.

## 5. Conclusions

In this study we obtained the first data on metabolome changes in the Siberian salamander *S. keyserlingii*. It belongs to Urodela (Caudata), while the rest of the freeze tolerant amphibians are anurans, so we would expect certain metabolic differences. The Siberian salamander was found as the only known amphibian not using glucose as the low molecular weight cryoprotectant; its role is played by glycerol. As expected, glycolysis was highly activated in the liver, but unexpectedly we found no accumulation of succinate expected in hypoxia. We observed high concentrations of the products of nucleic acid and biomembrane degradation. All this suggests that the Siberian salamander may employ distinct biochemical responses to freezing compared to other tolerant species.

## Figures and Tables

**Table 1:** Freezing protocol for the Siberian salamanders.

T, °C	Duration, Days
15	5
10	7
8	7
5	30
1	20
−1	20
−2	2
−3	2
−8	7

**Table 2:** Average concentrations of the detected metabolites in the organs of *S. keyserlingii* (nmoles per gram of wet tissue) ± standard error (*n* = 5); n/a, not detected. Statistical significance between frozen and control samples: * Welch test *p* < 0.05; ** *p* < 0.01; *** *p* < 0.001.

Compound	Liver		Muscle	
	Control	Frozen	Control	Frozen
2-aminoadipate	300 ± 175	142 ± 53 ***	252 ± 56	133 ± 25
3-aminoisobutyrate	3 ± 1	27 ± 2 ***	13 ± 3	26 ± 5
3-OH-isobutyrate	23 ± 4	539 ± 77 ***	32 ± 5	109 ± 20 **
Acetate	764 ± 19	232 ± 85 ***	121 ± 7	28 ± 4 ***
ADP	97 ± 23	25 ± 3 *	128 ± 32	42 ± 7*
Alanine	1009 ± 227	1973 ± 195 *	1915 ± 97	1099 ± 54 ***
Alpha-aminobutyrate	4 ± 1	10 ± 2 *	7 ± 1	4 ± 1
AMP	141 ± 13	18 ± 2 ***	88 ± 17	23 ± 13 *
Anserine	37 ± 5	210 ± 19 ***	41 ± 4	16 ± 4 **
Ascorbate	n/a	130 ± 21	n/a	n/a
Asparagine	38 ± 7	19 ± 4	130 ± 18	24 ± 9 ***
Aspartate	547 ± 163	264 ± 45	343 ± 76	66 ± 9 ***
ATP	78 ± 22	20 ± 6 *	102 ± 48	14 ± 5
Beta-alanine	160 ± 5	404 ± 38 ***	169 ± 24	395 ± 36 ***
Carnosine	20 ± 2	98 ± 25 *	1805 ± 373	940 ± 193
Choline	154 ± 25	475 ± 77 **	40 ± 6	41 ± 9
Citrate	27 ± 5	82 ± 7 ***	n/a	n/a
Creatine	101 ± 16	478 ± 59 ***	5525 ± 484	3544 ± 121 **
Ergothioneine	90 ± 20	322 ± 36 ***	24 ± 5	31 ± 6
Ethanol	50 ± 9	108 ± 24	n/a	n/a
Formate	32 ± 1	43 ± 3 *	163 ± 20	14 ± 5 ***
Fumarate	108 ± 12	58 ± 12 *	12 ± 2	18 ± 1
GABA	12 ± 2	24 ± 4 *	n/a	n/a
Glucose	5469 ± 1415	202 ± 87 **	738 ± 210	630 ± 136
Glutamate	2800 ± 149	1068 ± 271 ***	887 ± 118	508 ± 82 *
Glutamine	88 ± 15	73 ± 8	121 ± 11	119 ± 17
Glycerol	5721 ± 2161	200642 ± 19242 ***	5082 ± 1367	92500 ± 3211 ***
Glycerophosphocholine	779 ± 62	3920 ± 214 ***	873 ± 55	634 ± 22 **
GSH	171 ± 8	53 ± 9 ***	41 ± 25	1 ± 1
GTP	5 ± 1	11 ± 1 **	n/a	n/a
Histidine	44 ± 4	94 ± 13 **	53 ± 18	292 ± 30
Hypoxanthine	39 ± 5	280 ± 22 ***	21 ± 4	57 ± 8 ***
Inosinate	36 ± 8	7 ± 2 **	129 ± 33	166 ± 36 **
Inosine	8 ± 1	32 ± 5 **	24 ± 9	96 ± 25
Isoleucine	33 ± 4	89 ± 20 *	36 ± 7	51 ± 7 *
Lactate	878 ± 270	2268 ± 371 *	5705 ± 2273	2302 ± 158
Leucine	104 ± 13	273 ± 47 **	79 ± 17	133 ± 38
Lysine	122 ± 37	254 ± 44	n/a	n/a
Malic acid	809 ± 108	380 ± 58 **	n/a	n/a
Mannose	123 ± 39	20 ± 3 *	43 ± 7	1 ± 1 *
Me-Histidine	n/a	n/a	242 ± 74	128 ± 22
Methionine sulfoxide	20 ± 3	53 ± 14	n/a	n/a
Myo-inositol	10812 ± 493	19365 ± 967 ***	6219 ± 562	6155 ± 710
NAD	25 ± 5	40 ± 3 *	46 ± 12	11 ± 4 *
NADH	6 ± 2	n/a **	n/a	n/a
NADPH	4 ± 1	2 ± 1	n/a	n/a
Nicotinamide	21 ± 4	90 ± 3 ***	13 ± 8	4 ± 4
Ornithine	74 ± 10	174 ± 36 *	233 ± 24	157 ± 7 *
Phenylalanine	20 ± 5	56 ± 15	17 ± 4	52 ± 10 *
Phosphocholine	44 ± 8	62 ± 9	48 ± 9	24 ± 4 *
Pyruvate	4 ± 1	4 ± 1	23 ± 5	4 ± 1 **
S-adenosylhomocysteine	1 ± 1	10 ± 1 ***	n/a	n/a
Serine-phosphoethanolamine	n/a	n/a	3210 ± 249	1668 ± 132 ***
Scillo-inositol	73 ± 10	172 ± 18 **	159 ± 15	121 ± 15
Succinate	119 ± 27	103 ± 29	16 ± 2	8 ± 1 **
Threonine	91 ± 11	574 ± 50 ***	283 ± 50	273 ± 46
Tyrosine	20 ± 6	93 ± 19 **	15 ± 5	58 ± 9 **
UMP	20 ± 1	1 ± 1 ***	n/a	n/a
Uracyl	16 ± 2	25 ± 5	n/a	n/a
Uridine	9 ± 2	53 ± 11 **	n/a	n/a
Valine	119 ± 25	288 ± 48 *	61 ± 10	117 ± 15 *

## Data Availability

Not applicable.

## Supplementary Materials

The following are available online at https://www.mdpi.com/article/10.3390/biology10111172/s1, Figure S1: Heatmap for metabolite levels in liver of frozen and control Siberian salamanders. Figure S2: Heatmap for metabolite levels in hindlimb muscles of frozen and control Siberian salamanders.
