# Supplementary figures and images for: Biochemical Response to Freezing in the Siberian Salamander Salamandrella keyserlingii
Source: Biology (Basel). 2021 Nov 12;10(11):1172. doi: 10.3390/biology10111172 (PMC8614755; doi:10.3390/biology10111172)

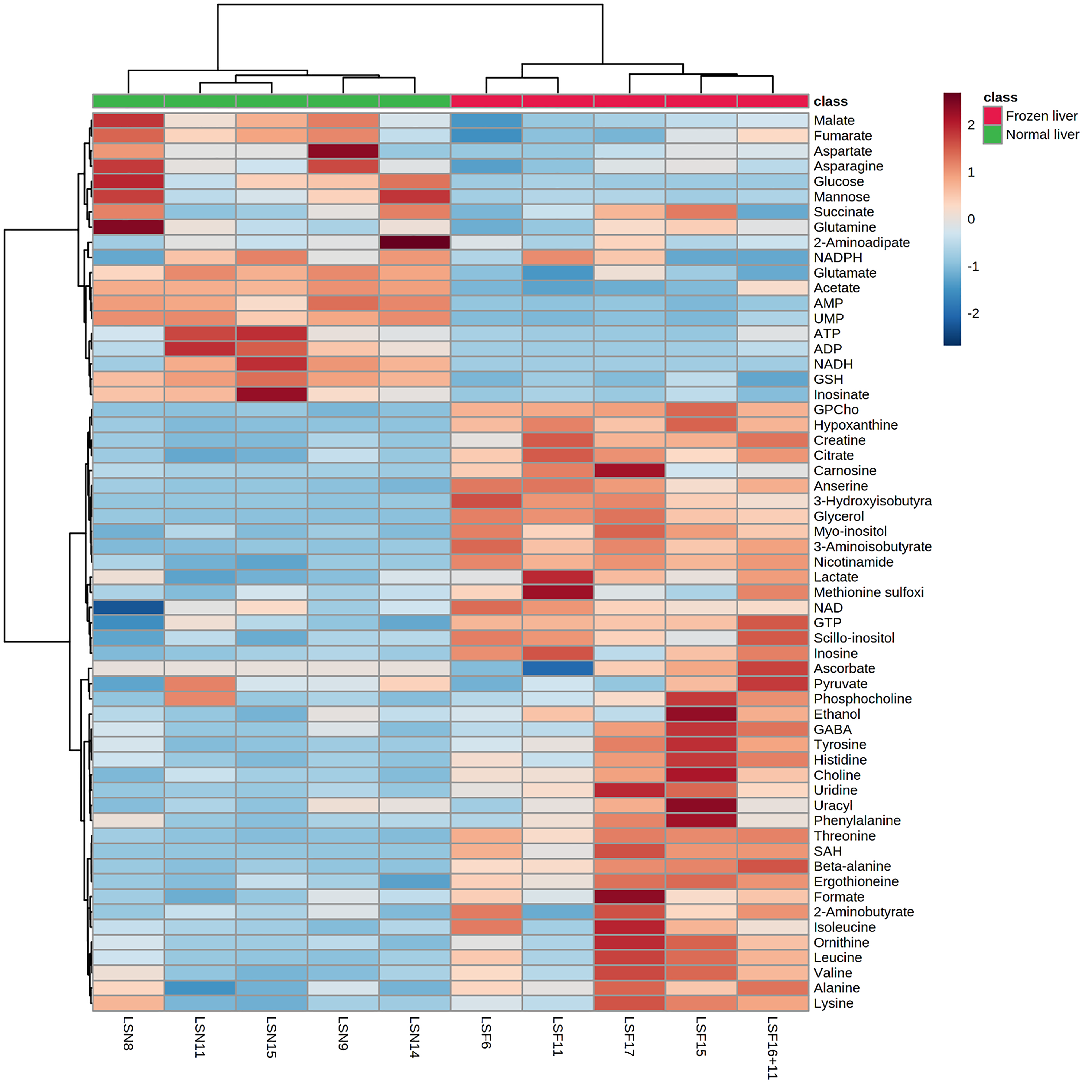

Supplement: Supplementary file 1 [file biology-10-01172-s001.zip › Suppl. Fig. 1.tif]

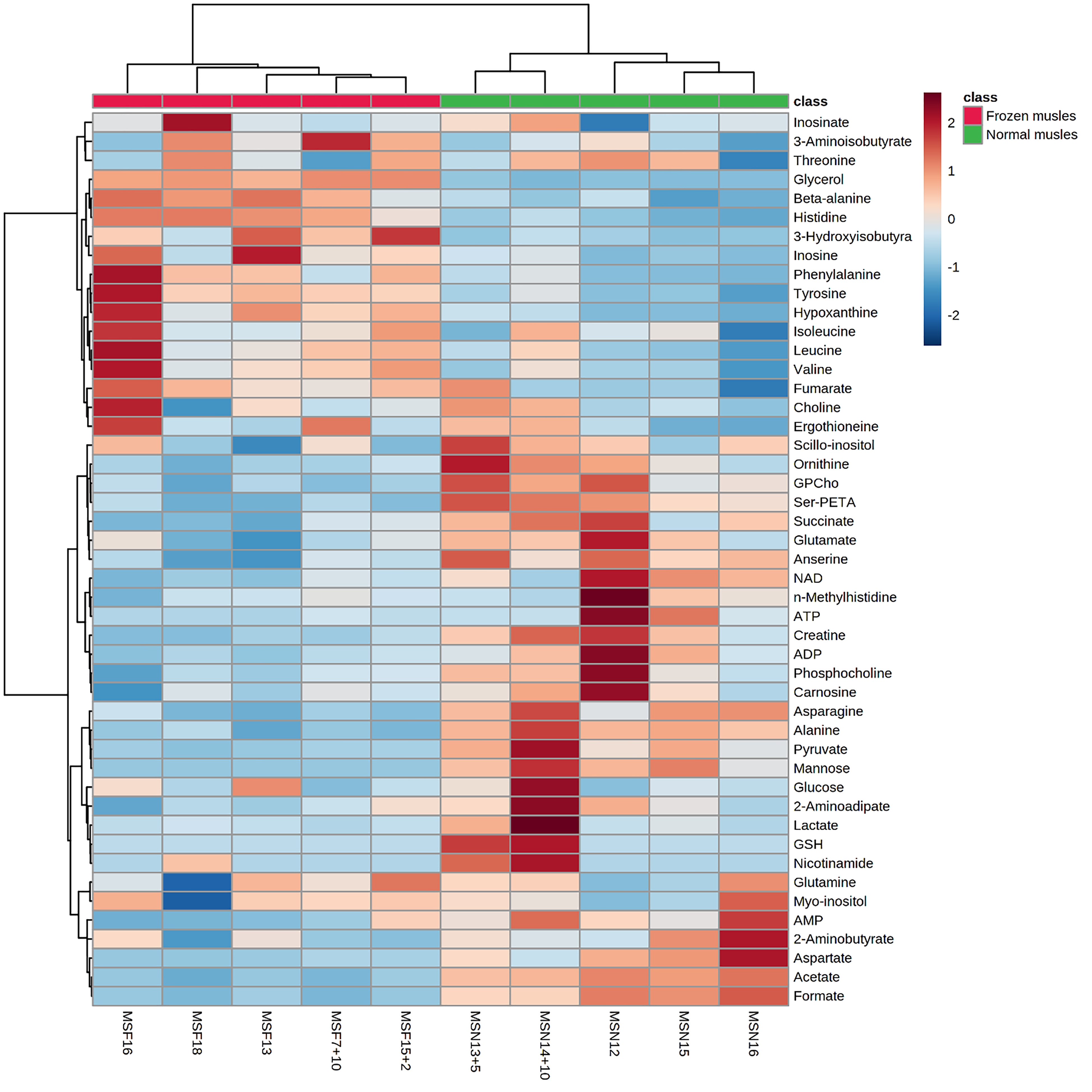

Supplement: Supplementary file 1 [file biology-10-01172-s001.zip › Suppl. Fig. 2.tif]
